# PDZ interaction of Vangl2 links PSD-95 and Prickle2 but plays only a limited role in the synaptic localisation of Vangl2

**DOI:** 10.1038/srep12916

**Published:** 2015-08-10

**Authors:** Tadahiro Nagaoka, Katsuhiko Tabuchi, Masashi Kishi

**Affiliations:** 1 Division of Cerebral Structure, Department of Cerebral Research, National Institute for Physiological Sciences, Okazaki 444-8787, Japan; 2 Department of Molecular and Cellular Physiology, Shinshu University School of Medicine, Matsumoto 390-8621, Japan

## Abstract

Postsynaptic density-95/Discs large/Zonula occludens-1 (PDZ) domain-mediated protein interactions play pivotal roles in various molecular biological events, including protein localisation, assembly, and signal transduction. Although the vertebrate regulator of planar cell polarity Van Gogh-like 2 (Vangl2) was recently described as a postsynaptic molecule with a PDZ-binding motif, the role of its PDZ interaction at the synapse is unknown. In this report, we demonstrate that the PDZ interaction was dispensable for the normal cluster formation of Vangl2 and not absolutely required for the synapse-associated localisation of Vangl2 in cultured hippocampal neurons. We further showed that the synaptic localisation of Vangl2 was categorised into two types: overlapping co-localisation with postsynaptic density (PSD)-95 or highly correlated but complementary pattern of association with PSD-95. Only the former was significantly sensitive to deletion of the PDZ-binding motif. In addition, the PDZ interaction enhanced the protein interactions between PSD-95 and Prickle2, which is another planar cell polarity factor that is localised at the postsynaptic density. Taken together with our recent report that the density of PSD-95 clusters was reduced in Vangl2-silenced neurons, these results suggest that Vangl2 determines the complex formation and clustering of postsynaptic molecules for synaptogenesis in mammalian brains.

The postsynaptic density (PSD) is an electron-dense disc-like organelle that is localised at the subsynaptic region of excitatory synapses and that contains crucial molecules for the reception of neurotransmitters^1^,^2^. These molecules include glutamate receptors, signaling enzymes, cytoskeletons and synaptic scaffolds, the mutual interaction of which is one of the most critical factors that determines the status of the individual synapse^1^,^3^. The Postsynaptic density-95/Discs large/Zonula occludens-1 (PDZ) domain is a protein interaction domain that is frequently found in multidomain-scaffolding proteins^4^ and that is contained by the excitatory postsynaptic scaffold protein, postsynaptic density-95 (PSD-95)^5^. PSD-95 is known to organize a protein complex of N-methyl-D-aspartate (NMDA)-type glutamate receptor channels by providing a link to other postsynaptic molecules, such as Shaker-subfamily potassium ion channels^6^, Neuroligin^7^, neuronal nitric oxide synthase^8^, TARPs^9^ and SynGAP^10^, through PDZ interactions. These interactions determine the localisation, trafficking and functional modulation of glutamate receptors and thereby play predominant roles in the regulation of synaptic plasticity^11^,^12^. Identifying molecules that interact with PSD-95 as well as elucidating the roles of their interactions will help improve our understanding of the detailed molecular mechanisms of learning and memory^13^.

Because neurons are among the most highly polarised cell types^14^, the regulators of cell polarisation are assumed to play central roles in the neuronal morphogenesis. In fact, we recently identified a vertebrate regulator of planar cell polarity (PCP), Van Gogh-like 2 (Vangl2), as a novel component of the PSD, which is required for the normal formation of dendritic spines^15^. Vangl proteins are orthologues of *Drosophila* Van Gogh/Strabismus (Stbm) tetraspanin^16^,^17^ which harbors a conserved carboxyl-terminal PDZ-binding motif (PBM: ETSV)^18^. This motif has been shown to bind *Drosophila* Discs large (Dlg)^19^ as well as its mammalian homologue PSD-95 but not other PDZ domain-containing proteins, such as Shank2^20^. Vangl2 plays an indispensable role in the normal clustering of PSD-95 in cultured hippocampal neurons^15^. Although PSD-95 is coimmunoprecipitated (co-IPed) with Vangl2 from synapse-rich brain extracts, the role of this protein interaction is still obscure^15^,^20^.

Transgenic rescue experiments of the *Drosophila Stbm* mutant have indicated that the PBM is not required for Stbm function^21^. Both Stbm-yellow fluorescent protein (YFP) fusion, in which the carboxyl-terminal PBM is masked by YFP, and Stbm ΔPBM, in which the PBM is simply deleted, are able to rescue the PCP phenotype of the *Stbm* mutant wings. However, in developing *Xenopus* embryos, the Stbm homologue lacking the PBM functions as a dominant-negative form of Stbm for the convergent extension movement, which suggests its important role^22^. Taken together, the requirements of the PBM seem to be context-dependent and therefore need to be analysed for each developmental event.

In the present study, we examined the roles of the PBM in the synaptic localisation of Vangl2 as well as in the complex formation of postsynaptic molecules. We found PBM-dependent and -independent mechanisms for the synaptic association of Vangl2. We further showed that PSD-95 and Prickle2 formed a protein complex through the carboxyl-terminal intracellular domain of Vangl2. Because Vangl2 without the PBM was able to form clusters and associate with the postsynaptic marker, Vangl2 might play a primary role in the organisation of postsynaptic molecules.

## Results

### Vangl2 links PSD-95 and Prickle2

**Figure 1:**
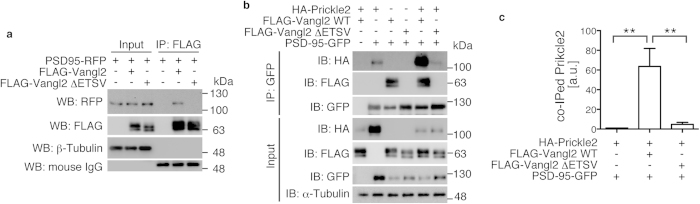
Interaction of Van Gogh-like 2 (Vangl2) with other postsynaptic density (PSD) proteins. (**a**, **b**) HEK293T cells were transfected with the indicated expression constructs, and the cell lysates as well as immuno-precipitates (IP) were analysed by western blotting (WB) with the indicated antibodies (Abs). IP was performed with either α-FLAG (**a**) or α-green fluorescent protein (GFP) (**b**) Abs. 0.5% of the cell lysate was loaded as Input. IB: immunoblot. (**c**) Bar graphs showing the precipitated ratio of Prickle2 in the WB analyses presented in (**b**). Note the robust enhancement of the complex formation between Prickle2 and PSD-95 by Vangl2 wild type (WT) but not by the ΔETSV. The amount of Prickle2 in the cell lysate was highly sensitive to the introduction of Vangl2^35^,^36^. The data are presented as mean ± standard deviation (SD). Significant differences (p < 0.05) versus control groups that were calculated with one-way ANOVA followed by Tukey’s multiple comparisons test are marked with *. **p < 0.005. a.u.: arbitrary unit.

Vangl2 is a tetraspanin with a carboxyl-terminal intracellular region (amino-acid residues 239–521 in mouse Vangl2), which contains both a Prickle/cadherin-binding domain (298–382)^15^,^23^ and the carboxyl-terminal PBM (ETSV; 518–521)^18^. Because Prickle2 directly binds to PSD-95 *in vitro*^24^, we examined whether the introduction of Vangl2 affected this interaction. The cDNA expression vectors of hemagglutinin (HA)-Prickle2 and green fluorescent protein (GFP)-PSD-95^25^,^26^ were cotransfected into HEK293T cells, and the cell lysate was subjected to immuno-precipitation (IP) with α-GFP antibodies (Abs) (Fig. 1b). On a western blot (WB) analysis of the IPs, we found a faint α-HA signal which indicated the PSD-95/Prickle2 interaction (Fig. 1b; 2nd lane). The introduction of the Vangl2 construct (FLAG-Vangl2) strongly increased the precipitated amount of Prickle2 (Fig. 1b; 5th lane, 1c; 63.6 ± 18.3-fold increase, n = 3), which indicated that the Prickle2/PSD-95 interaction was enhanced by Vangl2. In contrast, the deletion construct of Vangl2, which lacked the PBM and which was unable to co-precipitate PSD-95-red fluorescent protein (RFP) (Fig. 1a; 6th lane)^25^,^26^, did not greatly enhance the Prickle2/PSD-95 interaction (Fig. 1b; 6th lane, 1c), which suggested that this enhancement was mediated through the PDZ interaction between Vangl2 and PSD-95. In this experiment, the introduction of Prickle2 did not significantly enhance or inhibit the protein interaction between Vangl2 and PSD-95 (Fig. 1b; 3rd and 5th lanes), which suggested that the binding of PSD-95 to Vangl2 was independent of Prickle2. Similar results were also obtained in the WB analysis of α-HA IPs with α-GFP Abs (Figure S1). Taken together, these results suggest a role of Vangl2 in organizing the PSD molecules through the PBM.

### Vangl2 clusters and associates with the synapse without its PDZ interaction

In our previous WB analysis, we found that Vangl2 was highly enriched in the PSD fraction that was obtained by cell fractionation with density gradient ultracentrifugation of the mouse brain homogenates^15^. We further showed that α-Vangl2 Abs exhibited a punctate and synaptic pattern of immunofluorescence (IF) signals on the developed culture of hippocampal neurons as well as on the frozen sections of mouse brains^15^. In these experiments, however, aldehyde-based fixatives masked the epitope that was recognized by the Abs (Nagaoka and Kishi, unpublished observation). Thus, methanol fixation was required to obtain the synaptic staining. In order to observe the synaptic localisation of Vangl2 on the neurons with well-preserved plasma membranes, we introduced the amino-terminal (N-terminal) GFP-tagged construct of Vangl2, which was functional enough to rescue the phenotypes of the Vangl2-mutant mice^27^, into the cultured neurons and fixed them with paraformaldehyde. For each eye field of neuronal cultures, images of the GFP signals, as well as those of the α-PSD-95 signals, that were amplified with the secondary Abs that were labelled with red fluorophore were captured and digitally merged.

**Figure 2:**
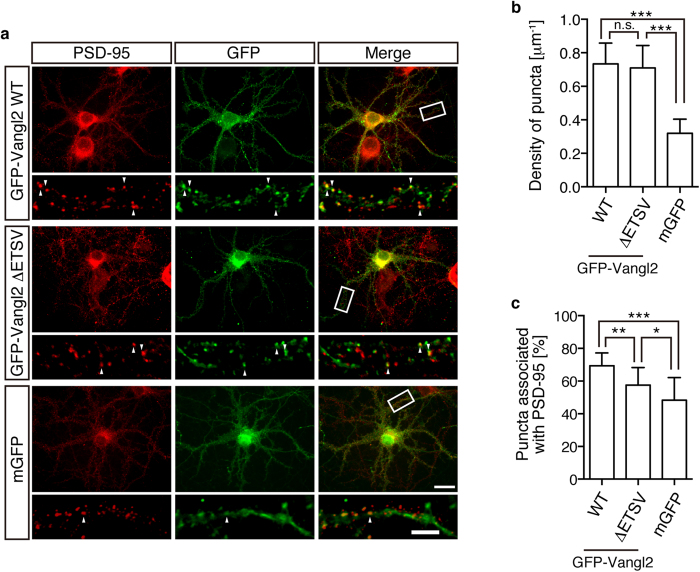
Punctate and synaptic localisations of Vangl2 with or without PDZ-binding motif. (**a**) Rat hippocampal neurons were transfected with the indicated expression plasmids (upper: GFP-Vangl2 WT, middle: GFP-Vangl2 ΔETSV, lower: mGFP) and cultured for 21 days. GFP fluorescence (green), α-PSD-95 immunofluorescence (IF; red) as well as their merged images are presented. Note the punctate localisation of GFP-Vangl2 ΔETSV and the mostly flat signals of mGFP. (**b**) Bar graphs showing the density of the GFP puncta that were formed by the indicated expression constructs along the dendrites. Note the similar levels of puncta formation between GFP-Vangl2 WT and ΔETSV. (**c**) Bar graphs showing the ratio of the PSD-95-associated population of formed GFP puncta for each expression construct. Note that the clusters of Vangl2 ΔETSV are more associated with PSD-95 than those of the simply membrane-tethered protein (mGFP). The highly magnified images of the delimited region are shown in the respective lower panels. Some examples of the co-localised puncta are indicated with arrowheads. The data are presented as mean ± SD. Significant (p < 0.05) and insignificant differences versus control groups that were calculated with one-way ANOVA followed by Tukey’s multiple comparisons test are marked with * and n.s., respectively. *p < 0.05, **p < 0.005, ***p < 0.0001. Scale bars: 20 μm and 5 μm for the upper and lower panels, respectively.

We first examined the requirement of the PBM in the cluster formation of Vangl2 in the cultured neurons. As reported previously, GFP-tagged wild-type Vangl2 (GFP-Vangl2 WT) showed clustering (0.73 ± 0.12 puncta/μm) with partial co-localisation (69.4 ± 7.8%) with the PSD-95 puncta on the dendrites (Fig. 2a; upper row)^15^,^20^. To our surprise, GFP-Vangl2 lacking the PBM (GFP-Vangl2 ΔETSV) also exhibited a punctate pattern of localisation (Fig. 2a; middle row), even without a significant reduction in the dot density (0.71 ± 0.13 puncta/μm; p = 0.787) (Fig. 2b). Because Vangl2 even without the PBM was assumed to be a membrane protein, it was possible that the clusters were formed only because they accumulated at the membrane-rich regions on the neuronal surface. In other words, GFP-Vangl2 ΔETSV may look clustered only because it is associated with membranes. In order to exclude this possibility, we examined the dot formation of membrane-associated GFP (mGFP) (Fig. 2a; lower row)^28^. Although we observed some puncta that were formed by mGFP, the density of these puncta along the dendrites was much less than that of the Vangl2-related constructs (0.32 ± 0.08 puncta/μm; p < 0.0001) (Fig. 2b). These results indicated that the PBM was dispensable for the normal cluster formation of Vangl2 on cultured hippocampal neurons.

Next, we examined whether the synaptic localisation of Vangl2 was affected by deletion of the PBM (Fig. 2c). Because the PBM of Vangl2 has been reported to be required for its normal colocalisation with PSD-95^20^, we quantified the ratio of the GFP-Vangl2 puncta that was associated with the PSD-95 clusters. Although we observed reduced co-localisation, punctate signals of GFP-Vangl2 ΔETSV were still significantly associated with PSD-95 (57.6 ± 10.7%; p = 0.0037) (Fig. 2c). In the control experiment, the formed clusters of mGFP were associated less with PSD-95 in ratio (48.3 ± 13.8%; p = 0.028 to GFP-Vangl2 ΔETSV), which excluded the possibility that the membrane association of GFP-Vangl2 ΔETSV was the determinant of its co-localisation with PSD-95. These results suggested that the PBM of Vangl2 was not absolutely required but facilitated the synaptic localisation of Vangl2.

### Complementary pattern of co-localisation of Vangl2 with PSD-95

**Figure 3:**
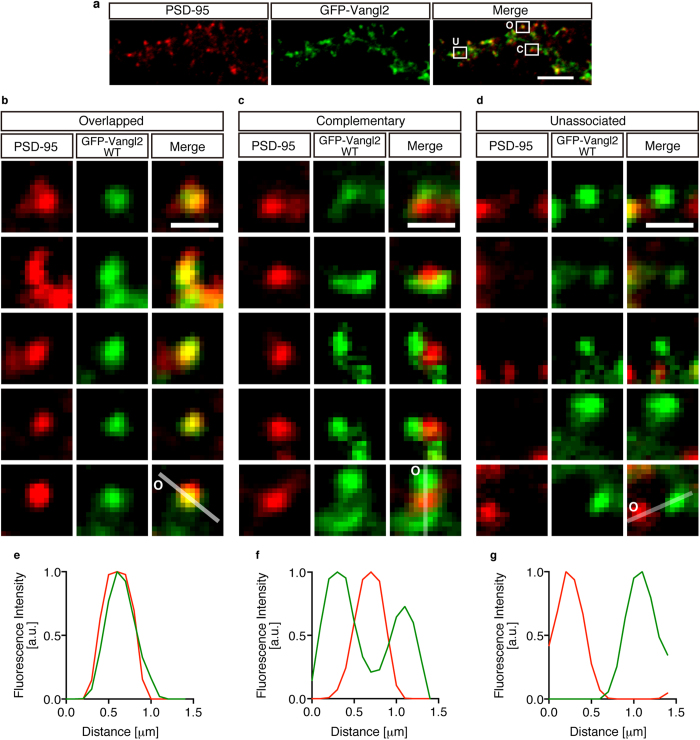
Identification of the three types of Vangl2 clusters in terms of association with PSD-95. (**a**) The overall distribution of GFP-Vangl2 WT puncta on the dendrites of electroporated neurons in culture. An example of each type of punctum is indicate and marked with a capitalized letter (O: overlapped, C: complementary, U: unassociated). (**b**,**c**,**d**) The highly magnified images of the GFP signals of each GFP-Vangl2 WT punctum (green) merged with those of the IF signals of PSD-95 (red). Some examples of puncta with overlapped (**b**), highly associated but complementary (**c**) and essentially unassociated (**d**) localisation are presented. The puncta delimited in (**a**) are shown in the upper most row. (**e**,**f**,**g**) Plot-profile analysis of one of the merged images shown in (**b**,**c**,**d**). The images in the lowest row were analyzed. Note that the PSD-95 signals occupy the hollow of the GFP signals in this particular example (**f**). “O”s and the white lines shown in (**b**), (**c**) and (**d**) indicate the origins and tracks of the plot-profile analyses presented in (**e**), (**f**) and (**g**), respectively. Scale bars: 1 μm.

While examining the highly magnified images of the GFP puncta, we realized that there were three types of Vangl2 clusters in terms of the co-localisation with PSD-95 (Fig 3a). The first was the overlapped co-localisation with PSD-95 (Fig. 3b). The second was the highly correlated but complementary association with PSD-95 (Fig. 3c), and the third was the almost complete unassociated localisation with PSD-95 (Fig. 3d). A representative example for each type (Figs 3b–d, lowest row) is depicted by the plot-profile analysis of the signal strengths of both fluorescences (Figs 3e–g for 3b, 3c and 3d, respectively). Although highly enriched in the PSD fraction as a whole, Vangl2 localised to the complementary regions that were associated with the PSD marker in a certain population of synapses. These results indicated that a spatiotemporal regulation of its localisation may play a role in the Vangl2 functions at the synapse.

### Synaptic localisation of Vangl2 by both PDZ interaction-dependent and -independent mechanisms

**Figure 4:**
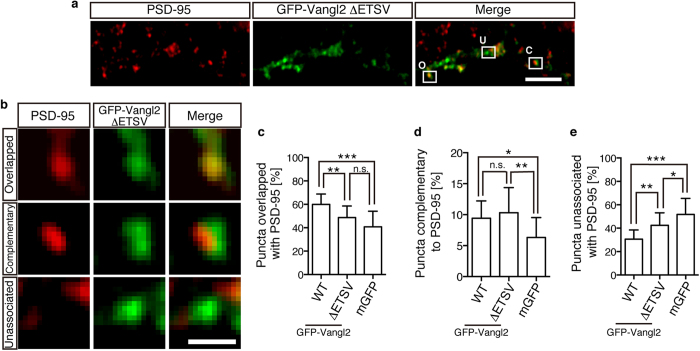
Statistical analysis of the synaptic localisations of Vangl2 with or without PDZ-binding motif. (**a**) Overall distribution of GFP-Vangl2 ΔETSV and PSD-95 puncta on the dendrites of electroporated neurons in culture. An example of each type of punctum is indicated and marked with a capitalized letter (O: overlapped, C: complementary, U: unassociated). (**b**) The highly magnified images of the indicated regions in (**a**), showing the overlapped, complementary and unassociated localisation with PSD-95 IF. (**c**) Bar graphs showing the ratio of the GFP puncta with overlapped co-localisation with PSD-95. Note the significant reduction in the overlapped ratio by the deletion of PBM (ΔETSV). (**d**) Bar graphs showing the ratio of GFP puncta with a complementary pattern of association with PSD-95. Note that the deletion of the PBM did not affect the occurrence of the complementary association. (**e**) Bar graphs showing the ratio of GFP puncta without association with PSD-95. Scale bars: 20 μm (**a**) and 5 μm (**b**). The data are presented as mean ± SD. Significant (p < 0.05) and insignificant differences versus control groups that were calculated with one-way ANOVA followed by Tukey’s multiple comparisons test are marked with * and n.s., respectively. *p < 0.05, **p < 0.005, ***p < 0.0001.

In order to reveal the role of the PBM in the finer localisation of Vangl2, we quantified the ratio of puncta with an overlapped or complementary association of GFP-Vangl2 with PSD-95 (Fig. 4a,b). Punctate signals of GFP-Vangl2 WT exhibited an average association of 69.4% with PSD-95 (Fig. 2c). Among them, 60.0 ± 8.8% were overlapped, and 9.4 ± 2.8% were complementary (Fig. 4c,d). By deleting the PBM, the ratio of the dots with overlapped co-localisation was significantly reduced (Fig. 4c; 47.3 ± 9.8%; p = 0.0016). The ratio was slightly more than that of mGFP (42.0 ± 13.7%) on average, but the difference was not statistically significant (p = 0.292). In contrast, the complementary association was not affected by the PBM deletion (Fig. 4d; 10.3 ± 4.1%; p = 0.690). Briefly, the reduced ratio of the PSD-95-associated puncta that were formed by GFP-Vangl2 ΔETSV (Fig. 2c) was due to a reduction of the overlapped co-localisation (Fig. 4c) and not to the complementary association (Fig. 4d). These results suggested that the PBM was required for the normal localisation of Vangl2 at the PSD but not for the synaptic association itself.

## Discussion

In this study, we demonstrated roles of the PDZ interaction that was mediated through the carboxyl-terminal end of Vangl2. PSD-95 and Prickle2, which are two of the known PSD proteins, are linked by Vangl2. An IF analysis of the GFP-Vangl2 puncta on the transfected neurons showed that approximately 10% of these puncta exhibited a complementary pattern of association with PSD-95. We further showed that the PDZ interaction of Vangl2 was not absolutely required for the cluster formation or complementary association but for the overlapped co-localisation with PSD-95. To the best of our knowledge, Vangl2 is so far the only molecule which is highly enriched in the PSD by cell fractionation and which exhibits a complementary association with the PSD marker in a substantial population of the synapses.

The similar complementary association with PSD has also been reported for N-cadherin^29^,^30^,^31^,^32^. In spines, N-cadherin is localised at the outer rim of the synaptic contacts, either circumventing the PSD or concentrated at the edges of the PSD. This localisation leads to the fine compartmentalisation of the postsynaptic areas, and it has been hypothesized to be responsible for preventing the diffusion of the synaptic molecules^33^,^34^. In our recent study, Vangl2 was described as a novel PSD molecule which is co-IPed with PSD-95 from the brain extracts. However, Vangl2 directly associates with N-cadherin to mediate normal spine formation through direct regulation of N-cadherin endocytosis^15^. Therefore, the two kinds of synaptic localisation of Vangl2 that were demonstrated in this study might reflect these protein interactions at the postsynapse.

In this regard, it is noteworthy that the binding of N-cadherin to Vangl2 was competitively inhibited by the PSD molecule Prickle2^15^. At least in part, whether synaptic Vangl2 localised to the PSD or its complementary regions may be determined by whether Vangl2 interacts with Prickle2 or with N-cadherin, respectively. In the former situation, the tertiary protein complex that is formed between PSD-95, Vangl2 and Prickle2, which is presented in this study (Fig. 1), may play a role in the establishment of the PSD. In *Drosophila*, Vang/Stbm recruits Prickle to the cell edges and also promotes proteasomal degradation of excess Prickle to generate normal PCP feedback and asymmetry^35^,^36^. Synaptic Vangl2 is therefore likely to control local protein level of Prickle2 (see Fig. 1b) and thereby determine the finer distribution of Prickle2 around the PSD. Because Vangl2 is a regulator in the development of cell polarity, whose *Drosophila* homologue is preferentially distributed to discrete membrane subdomains^37^, future studies on the control of these protein interactions and degradation will reveal the mechanism of the regionalisation of the postsynaptic areas.

The normal formation of the Vangl2 clusters even without the PDZ interaction suggests that Vangl2 is not clustered by PSD-95 on the cultured neurons. Considering our recent result that the clustering of the PSD-95 was reduced in the Vangl2-silenced neurons^15^, Vangl2 seems to play a role in clustering PSD-95. Similarly, during *Drosophila* embryogenesis, Stbm promotes the translocation of the PSD-95 homolog Dlg from the cytoplasm to membrane structures in a PBM-dependent manner^19^. The same protein interaction was also required for the anterior localisation of Dlg during the asymmetric cell division of the *Drosophila* sensory organ^38^. Interestingly, in the same context, Dlg forms a protein complex with Partner of Inscuteable (Pins)^39^, the mammalian homologue of which, mPins, enhances the trafficking of a PSD-95 paralogue SAP102 to the plasma membrane in order to mediate the cell surface expression of the NMDA receptors^40^. Future studies on the molecular interactions between Vangl2, mPins and PSD-95 at the PSD may reveal PCP signaling-mediated control of the development and functions of excitatory synapses.

## Methods

### Ethics statement

The experimental protocols were approved by the Institutional Review Board at the National Institute for Physiological Sciences.

### Chemicals

All chemicals were purchased from Nacalai Tesque, Inc. (Kyoto, Japan), Wako Pure Chemical Industries, Ltd. (Osaka, Japan) or Sigma-Aldrich Co. LLC (St. Louis, MO, USA) unless otherwise stated.

### Animals

The Animal Use and Care Committee authorized all of the animal experiments, which were performed in accordance with the NIH *Guidelines for Care and Use of Laboratory Animals*.

### Plasmids and antibodies

The expression constructs of FLAG-Vangl2 WT, GFP-Vangl2 WT and HA-Prickle2 were as described^15^,^41^. Fluorescent protein-tagged PSD-95 constructs were kind gifts from Dr. Choquet^25^,^26^. Deletion of the PBM was conducted by PCR-mediated introduction of a stop codon. mGFP was obtained from Addgene (#14757)^28^. The supplemental Excel file contains detailed information on the Abs used in this study. The plasmid constructs were sequenced to confirm the absence of sequence errors.

### Tissue culture

HEK293T cells (American Type Culture Collection, Manassas, VA, USA) were maintained in standard conditions with Dulbecco’s Modified Eagle’s Medium containing GlutaMAX (Life Technologies, Grand Island, NY, USA) that was supplemented with 10% foetal bovine serum (BioWest LLC, Kansas City, MO, USA) at 37 °C in the presence of 5% CO_2_ in a water-jacketed incubator (Thermo Fisher Scientific Inc., Waltham, MA, USA). Transfections of the plasmid DNA were performed with polyethylenimine (#23966; Polysciences, Inc., Warrington, PA, USA) according to the manufacturer’s instructions. The total DNA amount that was transfected was adjusted to 3.0 μg for each well of a 6-well plate with an empty pcDNA3.1(+) vector (Life Technologies). After 24 h of transfection, the cells were harvested for the biochemical analysis.

### Immuno-precipitation and WB analysis

The immuno-precipitation and WB analyses were performed essentially as described^15^,^41^. Cell lysis buffer (1% NP-40; 150 mM NaCl; 50 mM Tris-Cl, pH 7.5; 2 mM ethylenediaminetetraacetic acid and 10% glycerol, which was supplemented with 20 μg/mL leupeptin) was used for all of the steps of the cell lysis and bead washing, which were performed either on ice or in the cold room at 4 °C. Western Blotting Substrate Plus (Pierce) and the STAGE-5000 Imaging System (AMZ System Science) were used to acquire the chemiluminescent images. The signal intensity was calculated using ImageJ. The Protein-protein interactions that were determined with the co-IP technique were quantified according to the ratio of the Prickle2 amount of precipitated protein (IP) to that of the total (Input), which was further divided by the relative amount of precipitated PSD-95-GFP.

### Neuronal culture, immuno-fluorescence microscopy and image analysis

Culturing the rat hippocampal neurons, immuno-staining, and image analysis were performed essentially as described^15^,^42^,^43^. Transfections of the plasmid DNA were performed with Nucleofector (Lonza Group, Ltd., Basel, Switzerland)^44^,^45^. The neurons were fixed at 37 °C for 15 min with 4% paraformaldehyde in phosphate-buffered saline containing 30% sucrose and then permeabilised with 0.1% Triton X-100 in phosphate-buffered saline for 20 min before applying the Abs. To count the molecular clusters, fluorescence puncta that had their major axes within 0.4–1.5 μm were taken into account. For each neuron, the number of punctate signals along dendrites with lengths that were at least 85 μm, was analysed. The average length of the analysed dendrites was 122 μm. For the statistical analysis, the counts of 20 neurons were averaged for each experiment (n = 3). For a plot-profile analysis of the signal intensity of both fluorescence along lines, ImageJ was used to quantify the intensity of each plot (0.1 μm).

### Statistical analysis

All of the counts and measurements were conducted in blind fashion. Each experiment was repeated at least three times and essentially produced consistent results. The measured values from three independent experiments were used for each statistical analysis. The average ratio of one of the samples was set as one arbitrary unit in order to represent the relative amounts, and this was used as the standard in the statistical analysis.

## Additional Information

**How to cite this article**: Nagaoka, T. *et al.* PDZ interaction of Vangl2 links PSD-95 and Prickle2 but plays only a limited role in the synaptic localisation of Vangl2. *Sci. Rep.* **5**, 12916; doi: 10.1038/srep12916 (2015).

## Supplementary Material

Supplementary Information

Supplementary Dataset 1
