# Supplementary material for: PDZ interaction of Vangl2 links PSD-95 and Prickle2 but plays only a limited role in the synaptic localisation of Vangl2
Source: Sci Rep. 2015 Aug 10;5:12916. doi: 10.1038/srep12916 (PMC4530445; doi:10.1038/srep12916)
Supplement: Supplementary Information [file srep12916-s1.docx]

**SUPPLEMENTARY INFORMATION**

**Supplementary Figure S1-2**

**PDZ interaction of Vangl2 links PSD-95 and Prickle2 but plays only a limited role in the synaptic localisation of Vangl2**

Tadahiro Nagaoka^1^, Katsuhiko Tabuchi^1,2^, and Masashi Kishi^1,*^

^1^Division of Cerebral Structure, Department of Cerebral Research, National Institute for Physiological Sciences, Okazaki 444-8787, Japan

^2^Department of Molecular and Cellular Physiology, Shinshu University School of Medicine, Matsumoto 390-8621, Japan

^*^correspondence: [masashi.kishi@gmail.com](mailto:masashi.kishi@gmail.com)

**Supplementary Figure S1: Interaction between PSD-95 and Prickle2 is enhanced by Vangl2**

HEK293T cells were transfected with the indicated expression constructs, and the cell lysates as well as IPs were analysed by WB with the indicated Abs. IP was performed with α-HA Abs. 0.5% of the cell lysate was loaded as “Input”.

**Supplementary Figure S2: Uncropped WB images**

(a, b, c) The original WB images used for Figure 1a (a), Figure 1b (b) and Figure S1 (c) are presented.
